# Effects of a Single Session of Systemic Vibratory Therapy on Flexibility, Perception of Exertion and Handgrip Strength in Chronic Obstructive Pulmonary Disease Individuals: A Quasi-Experimental Clinical Trial

**DOI:** 10.3390/jcm12093241

**Published:** 2023-05-01

**Authors:** Eliane de Oliveira Guedes-Aguiar, Redha Taiar, Laisa Liane Paineiras-Domingos, Bruno Bessa Monteiro-Oliveira, Danúbia da Cunha de Sá-Caputo, Mario Bernardo-Filho

**Affiliations:** 1 Laboratório de Vibrações Mecânicas e Práticas Integrativas, Departamento de Biofísica e Biometria, Instituto de Biologia Roberto Alcantara Gomes, Policlínica Universitária Piquet Carneiro, Universidade do Estado do Rio de Janeiro, Rio de Janeiro 20950-003, Brazil; 2 Programa de Pós-Graduação em Ciências Médicas, Faculdade de Ciências Médicas, Universidade do Estado do Rio de Janeiro, Rio de Janeiro 20551-030, Brazil; 3 MATériaux et Ingénierie Mécanique (MATIM), Université de Reims Champagne-Ardenne, 51100 Reims, France; 4 Departamento de Fisioterapia, Instituto Multidisciplinar de Reabilitação e Saúde, Universidade Federal da Bahia, Salvador 40210-905, Brazil; 5 Programa de Pós-Graduação em Fisiopatologia Clínica e Experimental, Faculdade de Ciências Médicas, Universidade do Estado do Rio de Janeiro, Rio de Janeiro 20551-030, Brazil

**Keywords:** whole-body vibration exercise, functionality, chronic obstructive pulmonary disease

## Abstract

Background: Whole-body vibration exercises (WBVE), that are generated in systemic vibratory therapy (SVT), may benefit individuals with chronic obstructive pulmonary disease (COPD). This study evaluated acute effects of SVT on the flexibility, on the perception of exertion to perform the anterior trunk flexion (ATF), and on the handgrip strength (HG). Methods: Thirty-eight individuals, separated into two groups, performed a single session of SVT (five bouts, 25 Hz, 2.5 of amplitude) on a side-alternating vibrating platform (SAVP), in two postures: sitting (Sitting group-SitG, *n* = 21) or standing (Stand group-StandG, *n* = 17). In both positions, the feet were on the base of the SAVP. The HG and the AFT were performed before and after the session, and the perception of effort (RPE) was measured during the ATF. Results: The ATF in the SitG (*p* ≤ 0.05) and in the StandG (*p* ≤ 0.05) was significantly improved, but in the comparison between both groups, no significant reduction was found (*p* = 0.14). The RPE was not influenced by the session. A significant increase of the HG in StandG post session (33.49 ± 10.30 kgf) *p* = 0.03 was found, but not in the SitG (*p* = 0.12) or between the two groups (*p* = 0.55). Conclusions: SVT, in a single acute session, would be capable of promoting some functional benefits for the COPD individuals without altering the perception of exertion to perform the ATF. Trial Registration: 49219115.3.0000.5259, RBR-72dqtm.

## 1. Introduction

Chronic obstructive pulmonary disease (COPD) is a pulmonary disease characterized by persistent airflow limitation [1], and extrapulmonary symptoms (systemic effects) lead to comorbidities and may contribute to the severity of the COPD [2]. The comorbidities due to the systemic effects might be loss of muscle mass, which, in turn, leads to inactivity, physical deconditioning [3], and lower limb muscle function impairment [4].

It is suggested that a decrease in muscle mass leads to a reduction in quadriceps femoris muscle strength, which is related to disuse and consequently to muscle atrophy [5]. Muscle atrophy is one of the main causes of decreased skeletal muscle strength, and lower limb circumference can predict mortality from COPD [6,7]. Moreover, decreased skeletal muscle strength and endurance exist in patients with mild COPD, even before the presence of respiratory symptoms [8].

A loss of flexibility in the lower limbs is also associated with COPD and is reflected in very rigid or very irregular movement patterns [9]. It is described that there is a reduction of muscle strength and functionality in elderly with COPD, a consequent increased risk of fall, and an intolerance to exercise leading to the adoption of a sedentary lifestyle [10].

Considering the physical frailty observed by Abdulai et al. 2018 [11] in COPD individuals, rehabilitation is beneficial and serves as an essential component in the management of COPD, since it improves health-related quality of life and exercise capacity [5,12]. Pulmonary rehabilitation (PR) involves various kinds of interventions, such as exercise. PR brings clinical improvements, such as the exercise capacity and skeletal muscle function of COPD sufferers [13], relief from dyspnea and fatigue, and an increased sense of disease control. However, many COPD individuals have a fear of exacerbating dyspnea during the PR [14]. In PR, different stretching strategies and protocols have been used to enhance flexibility or maintain health, acting on the muscle tendon–unit to improve the range of motion of the joints [15].

One type of exercise on the PR is the whole-body vibration exercise (WBVE), which has been suggested to COPD individuals [16,17]. WBVE is generated in an individual in the systemic vibratory therapy (SVT) [18,19,20] when mechanical vibration produced in a vibrating platform (VP) is transmitted to an individual [21] who is in contact with the base of the PV, which is turned on [22]. Acute [23] and cumulative effects [24] of SVT have improved the flexibility and rating of perceived exertion [25], lower limb strength and power [26], muscle tone and hamstring flexibility [27], and metabolic parameters [28,29]. SVT also leads to sequential muscle contraction and relaxation [30] that can lead to a maximum voluntary muscle strength that can be measured by the handgrip strength (HG) [31].

Zhou J. et al. 2018 [32] in a systematic review and meta-analysis investigated if the WBVE may be used to treat COPD patients. It was verified that WBVE has beneficial effects for COPD patients when compared with the control group. An increased 6 min walking distance (6-MWD) (*p* < 0.001), the reduction of the time to finish the five repeated sit-to-stand tests (*p* = 0.04), and improvement of St George’s Respiratory Questionnaire score (*p* < 0.001) were found.

Cardiorespiratory responses were used to investigate effects of WBVE with different mechanical vibration frequencies and two types of squatting exercises (static and dynamic) in COPD individuals. A decrease in the ratio of minute ventilation to oxygen production ($\dot{V}$e/$\dot{V}$o2), and in the ratio of minute ventilation to carbon dioxide production were found during static and dynamic squats. There was a significant difference with a reduction in oxygen saturation (40 Hz when compared with 30 Hz). The WBVE represented in this study, a mild effort that promoted cardiorespiratory response in COPD, had no adverse effect [33].

Gloeckl, et al., 2021 [34] pointed out that the WBVE improved physical performance in COPD individuals, considering the static balance performance (*p* = 0.032), the muscular power (*p* = 0.001), the 6-MWD test (*p* < 0.001), and the 4 m gait speed test (*p* = 0.018), compared with a control group.

The current study aimed to determine acute effects of SVT on the range of motion using the anterior trunk flexibility (ATF) test, on the perception of exertion across the Borg scale, and on the muscle strength across the handgrip strength in COPD elderly individuals. The hypothesis of this study was that the SVT could improve the functionality of COPD individuals.

## 2. Materials and Methods

### 2.1. Study Design

This is a quasi-experimental clinical trial. The recruitment of participants was carried out at Departamento de Pneumologia, Policlínica Universitária Piquet Carneiro (PPC), Universidade do Estado do Rio de Janeiro, Brazil and the clinical evaluation was performed by a medical staff. The subjects were referred to the Laboratório de Vibrações Mecânicas e Práticas Integrativas (LAVIMPI), also at PPC, to perform the protocol with SVT.

Inclusion and exclusion criteria were determined. The inclusion criteria were COPD individuals (i) of both gender, (ii) aged over 40 years old, (iii) being followed up at Departamento de Pneumologia of PPC with a diagnosis of COPD, (iv) stable (no exacerbation of the respiratory symptoms), (v) independent (able to stand on their own on the basis of the VP), and (vi) who signed the informed consent form (ICF).

The exclusion criteria were individuals with (a) exacerbation for less than three months, (b) presence of labyrinthitis, (c) reported osteoporosis, (d) other respiratory diseases, (e) use of a pacemaker, (f) previous history of fractures and/or other orthopedic diseases, (g) surgeries with implantation of metallic material, (h) peripheral vascular disease and/or thromboembolism, (i) severe tabagism and/or alcoholism, (j) decompensated cardiovascular disease, (k) aneurysm, (l) previous vitreous hemorrhage, (m) malnutrition, (n) recent postoperative period, (o) neurological disease that generates “fear” of movements in the VP, (p) severe or disabling clinical disease, and (q) at the discretion of the investigator.

### 2.2. Participants

**Figure 1:**
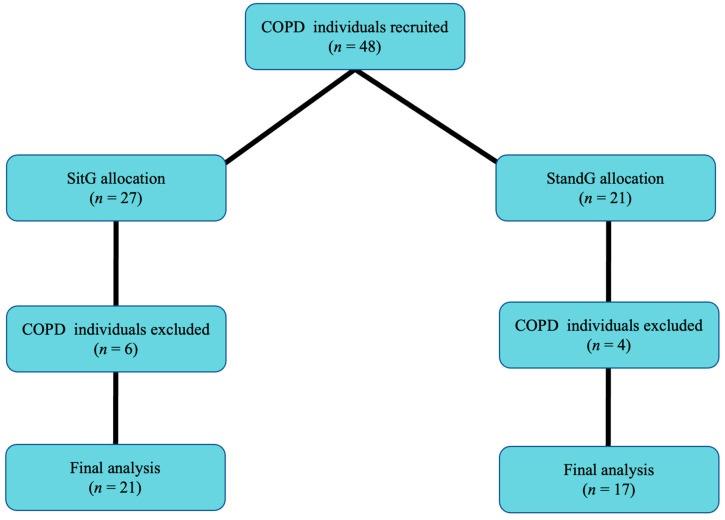
Flow of participants through the study. Sitting group (SitG); Stand group (StandG).

Forty-eight COPD individuals were recruited. Thirty-eight concluded the acute SVT protocol and were allocated to two different postures on the SAVP: SitG (*n* = 21) e StandG (*n* = 17). Ten COPD individuals gave up for personal reasons (Figure 1).

### 2.3. Study Protocol (Procedures)

On the first day, the COPD individuals signed the ICF before any session. Personal data obtained in the anamnesis were collected: age, gender, tabagism, diabetes mellitus type 2 (DMT2), systemic arterial hypertension (SAH), time of illness diagnosis of COPD, other associated diseases, use of medications, physical inactivity, and family history. Functional tests and other clinical information were collated.

On the second day clinical and anthropometric data were collected before the session: height, body mass, body mass index (BMI), with bioimpedance device (InBody 370) partial oxygen saturation (pSO_2_), and respiratory rate (RR) with portable digital oximeter (G-tech). After this, the COPD individuals perfomed the acute session of the SVT protocol.

HG is a simple and commonly applied test to assess the person’s general strength level. HG can be used to predict the risk of cardiovascular disease and all-cause mortality in the general population [35], and also in COPD individuals [36]. HG strength was measured as proposed by American Society of Hand Therapists [37].

**Figure 2:**
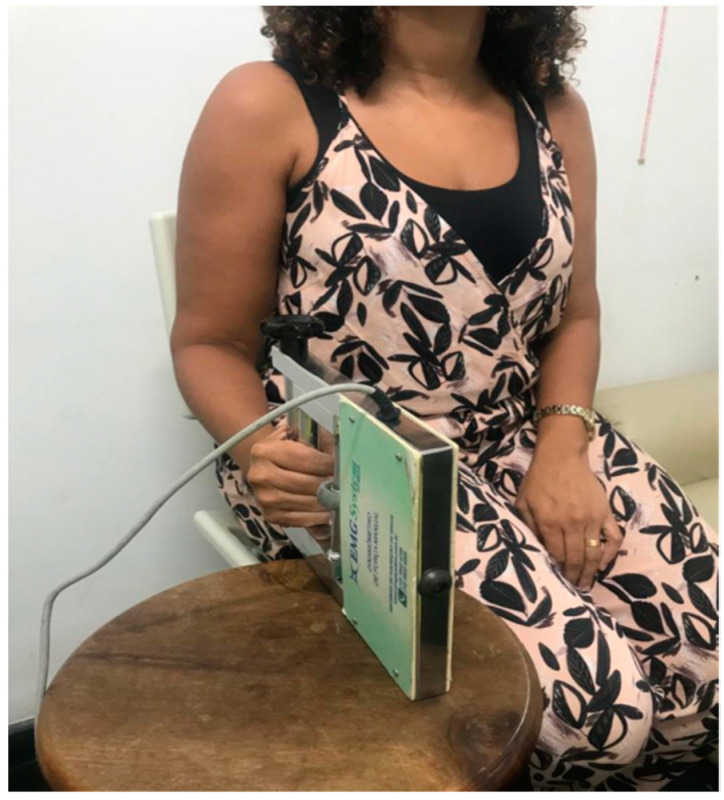
COPD individual sitting in a chair with arm in adduction, with 90° forward bend at elbow joint, forearm in neutral position, wrist with extension between 0° and 30°, and ulnar flexion between 0° and 15°.

The individuals were in the seated position, arms in adduction, bent 90° forward at the elbow joint, forearm in neutral position, wrist with extension between 0° and 30°, and ulnar flexion between 0° and 15°. Subjects performed three maximum attempts with a manual dynamometer (EMG832WF, EMG System, São José dos Campos/SP) with the right hand, for 6 s each, verbal encouragement, and 30 s of rest. (Figure 2).

COPD individuals performed a single SVT acute session. The SVT protocol consisted of 5 bouts with a 1 min working time and resting time of 1 min after each working time, making a total of 25 min per session. Mechanical vibration with frequency of 25 Hz and peak-to-peak displacement of 2.5 mm on a side-alternating vibrating platform (SAVP) (Novaplate^®^ Fitness Evolution, São Paulo, Brazil) was used.

**Figure 3:**
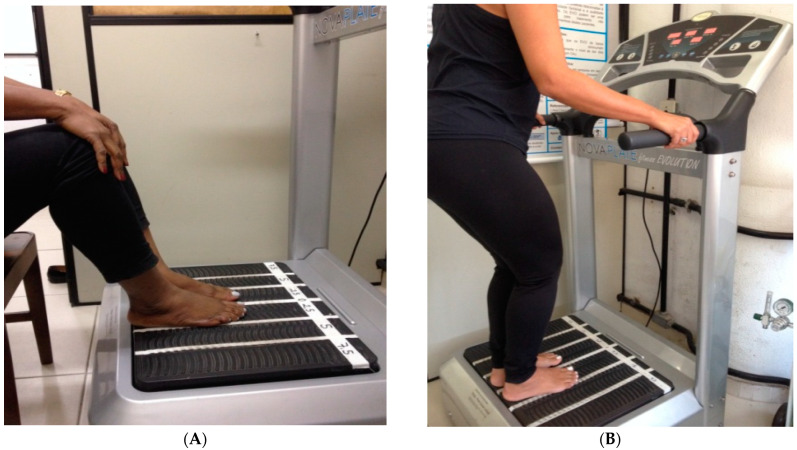
(**A**) COPD individual sitting in a chair with the hands maintained in contact with the knees and the feet on the base of the platform. (**B**) COPD individual standing on the SAVP, with the feet on the base of the platform.

The COPD individuals of the Sitting group (SitG) were sitting in an ancillary chair (Figure 3A) in front of the VP with the feet on the base of the VP and the hands maintained in contact with their knees. The individuals of the Stand group (StandG) were standing on the base of the SAVP (Figure 3B). The individuals in both groups had a squat position with the knees flexed at 120–130° (measured using a goniometer Carci^®^, São Paulo, Brazil).

All procedures were supervised by a physiotherapist who followed the procedures and instructed the patient to report any discomfort.

**Figure 4:**
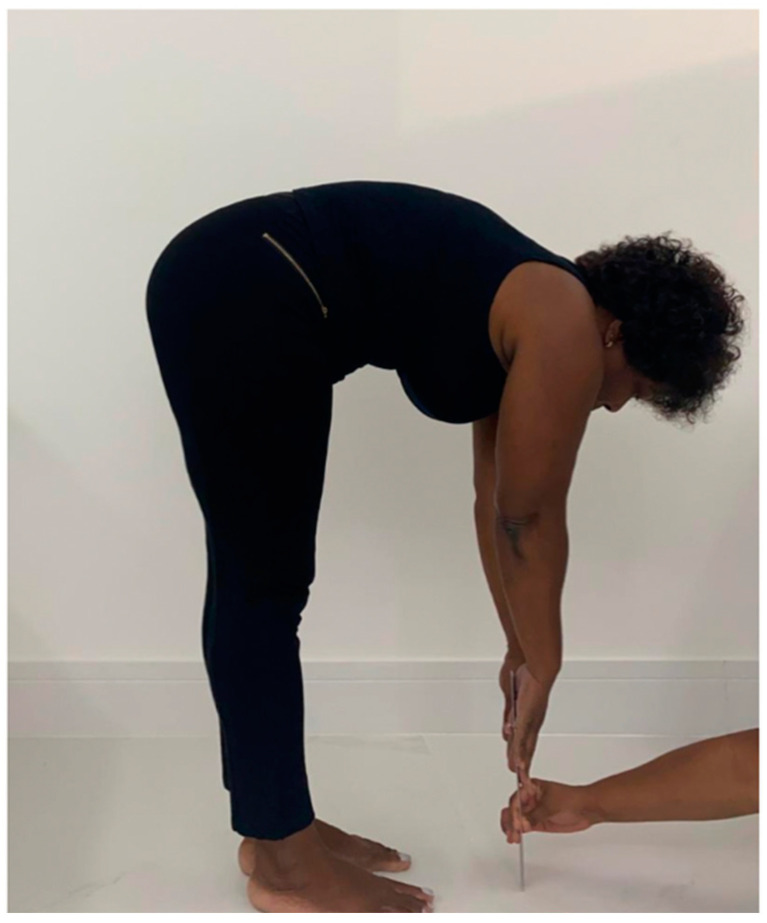
COPD individual perfoming the anterior trunk flexion to measure the range of motion of the trunk (flexibiity).

The range of motion of the trunk in the anterior trunk flexibility evaluation was measured by the ATF test [30,38] that is also called the fingertip-to-floor distance (FTFD) test [39]. This test consisted in measuring the distance between of the tip of the third finger and the floor, expressed in centimeters, after an anterior trunk flexion, with feet together, without bending the knees [38]. The test was applied before and after the SVT session (Figure 4).

The Rating of Perception of Effort (RPE) scale is a numerical scale from 6 to 20, ranging from “mild” (6 points) to “maximum effort” (20 points) [40] and aims to assess the individual’s perception of exertion, before and after the session of SVT [25].

The strength of muscles were analyzed across the HG strength of the right hand, using a dynamometer (EMG832WF, EMG System, São José dos Campos, Brazil), with standardized positioning and instruction [41,42]. The COPD individuals were in an upright position with the shoulder adducted and neutrally rotated, forearm in neutral position, and wrist between 0° and 30° dorsiflexion and between 0° and 15° of ulnar deviation. Three trials were performed with a 60-s rest in between and the average of the values (kgf) was used for the analysis [43].

### 2.4. Statistical Analysis

The sample size was calculated considering a type I error (α) of 5% and a type II error (β) of 20%. A standard deviation of the observed difference before and after the session of 2.5 was also considered, and the minimum difference to be detected was 2 (hypothetically defined according to preliminary results). Sixteen individuals were calculated for each group [44]. Data were entered and stored using Microsoft^®^ Excel 365. The statistical analysis was performed using GraphPad Prism 6.0. The Shapiro–Wilk test was used to evaluate the normality of the data. Confirming the normality of data, a paired Student t test was performed (intragroup). A Mann–Whitney U test was used for intergroup analysis. Pearson correlation analysis was performed. The characteristics of the participants were summarized using means and standard deviations (SD) or frequencies and percentages, as appropriate. For categorical variables, the Chi-square test was used. The significance level of *p* ≤ 0.05 was considered.

## 3. Results

### Individual Characteristics

The anthropometric characteristics and clinical data of the COPD individuals of both groups in the baseline are presented in Table 1. No differences were found between the groups.

About the range of motion of the trunk (ATF), a significant reduction of FTFD was found for the SitG, pre-session (19.62 ± 4.88 cm) and post session (17.29 ± 5.38 cm), *p* < 0.05. It was also found in the StandG, pre-session (16.55 ± 10.50 cm) and post session (13.41 ± 10.16 cm), *p* < 0.05. However, comparing both groups, no significant reduction was found (*p* = 0.14).

The RPE was measured during the ATF. No significant difference was found in both the paired groups (SitG—9.38 ± 3.26 × 8.61 ± 2.95, *p* = 0.13 and StandG—8.29 ± 3.07 × 8.05 ± 2.90, *p* = 0.62), or between the groups, (*p* = 0.48).

**Figure 5:**
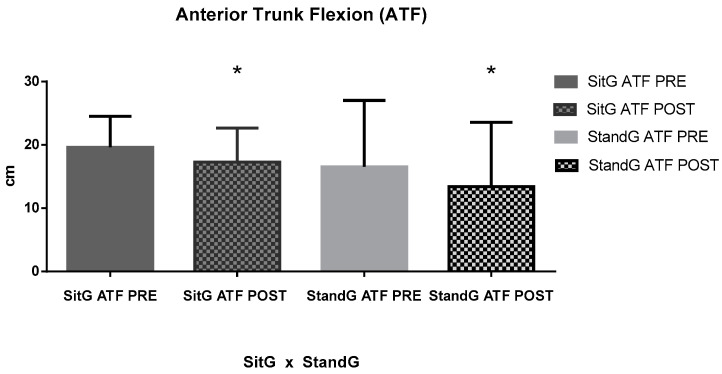
Anterior trunk flexion (ATF); Sitting group (SitG); Stand group (StandG). ATF measure of the Sitting group in pre-session (SiTG ATF PRE); ATF measure of the Sitting group in post-session (SiTG ATF POST); ATF measure of the Standing group in pre-session (StandG ATF PRE); ATF measure of the Standing group in post-session (StandG ATF POST). * *p* ≤ 0.05 pre × post session.

Analyzing the HG in the SitG pre-session (30.24 ± 8.44 kgf) and post session (31.61 ± 8.91 kgf), *p* = 0.12, no significant increase was observed. Comparing the StandG pre-session (28.64 ± 8.46 kgf) and post session (33.49 ± 10.30 kgf), *p* = 0.03, a significant increase of the HG was found (Figure 5).

**Figure 6:**
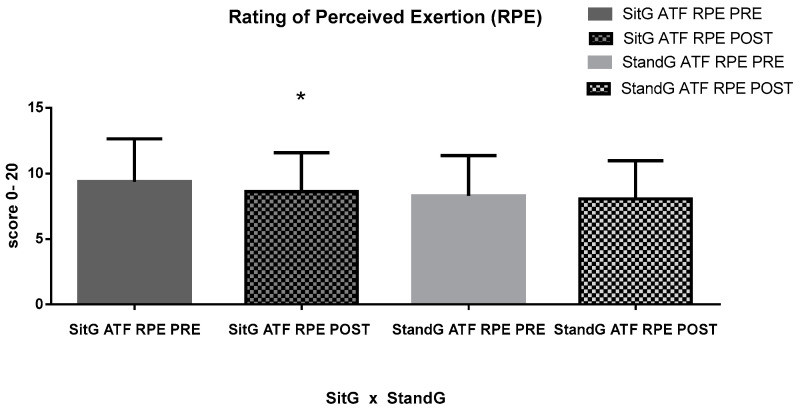
RPE of the ATF. Rating of Perceived Exertion (RPE); ATF—anterior trunk flexion (ATF); Sitting group (SitG); Stand group (StandG). Rating of Perceived Exertion of the anterior trunk flexion by the Sitting group during the pre-session (SitG ATF RPE PRE); Rating of Perceived Exertion of the anterior trunk flexion by the Sitting group during the post-session (SitG ATF RPE POST); Rating of Perceived Exertion of the anterior trunk flexion by the Standing group during the pre-session (StandG ATF RPE PRE); Rating of Perceived Exertion of the anterior trunk flexion by the Sitting group during the post-session (StandG ATF RPE POST). * *p* ≤ 0.05 pre vs. post session.

Pearson correlation analysis (r) between HG parameters and the ATF and the RPE of the ATF was performed. Considering the correlation between ATF and HG, SitG presented a negative correlation (r = −0.17, *p* = 0.46) while the StandG presented a positive correlation between these variables (r = 0.34, *p* = 0.17). The correlation analysis between RPE and ATF showed a positive correlation for SitG (r = 0.20, *p* = 0.37) and negative for StandG (r = −0.26, *p* = 0.30). A negative correlation was observed between the RPE and HG in SitG (r = −0.002, *p* = 0.99), while a positive correlation was found in StandG (r = 0.13, *p* = 0.61) (Figure 6).

**Figure 7:**
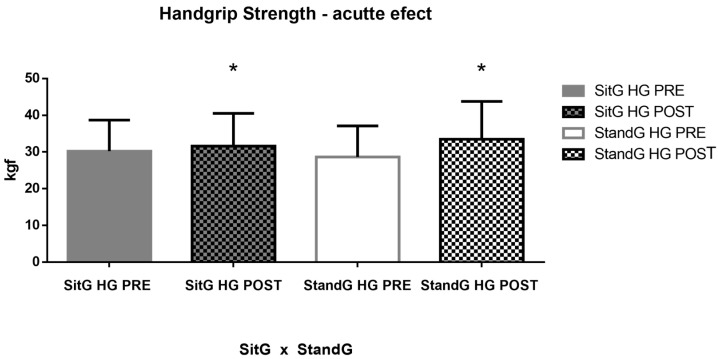
Acute effects of the SVT on the muscle strength by the HG. Handgrip (HG); Sitting group (SitG); Stand group (StandG). Handgrip measure of the Sitting group at the pre-session (SitG HG PRE); Handgrip measure of the Sitting group at the post-session (SitG HG POST); Handgrip measure of the Standing group at the pre-session (StandG HG PRE); Handgrip measure of the Standing group at the post-session (StandG HG POST). * *p* ≤ 0.05 pre vs. post session.

Figure 7 shows the muscle strength of the COPD individuals in both groups, across the HG measures. Analyzing the SitG pre session (30.24 ± 8.44 kgf) vs. the SitG post session (31.61 ± 8.91 kgf), *p* = 0.12, no significant increase of the HG is observed. However, comparing the StandG pre session (28.64 ± 8.46 kgf) vs. StandG post session (33.49 ± 10.30 kgf), *p* = 0.03, a significant increase of the HG was found. The unpaired *t*-test showed that no significant difference was observed between the two groups (*p* = 0.55).

**Figure 8:**
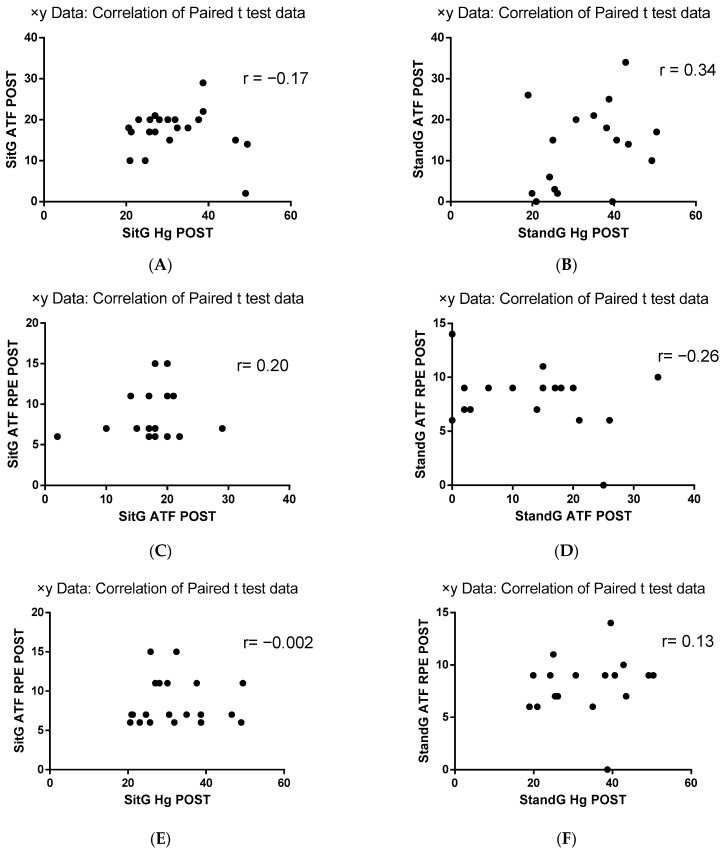
Pearson correlation (r) analysis. RPE—Rating of Perceived Exertion; ATF—anterior trunk flexion (ATF); Handgrip strength (HG); Sitting group (SitG); Stand group (StandG). (**A**) Pearson correlation of ATF × HG of the SitG. (**B**) Pearson correlation of ATF x HG of the StandG. (**C**) Pearson correlation of ATF × ATF RPE of the SitG. (**D**) Pearson correlation of ATF × ATF RPE of the StandG (StandG ATF POST). (**E**) Pearson correlation of ATF x HG of the SitG. (**F**) Pearson correlation of ATF × HG of the StandG.

Pearson correlation analysis (r) between HG parameters and the ATF and the RPE of the ATF was performed and is represented in Figure 8A–F. Considering the correlation between ATF and HG (Figure 8A,B), SitG presented a negative correlation (r = − 0.17, *p* = 0.46) while the StandG presented a correlation between these variables (r = 0.34, *p* = 0.17). The correlation analysis between RPE and ATF (Figure 8C,D) showed a positive correlation for SitG (r = 0.20, *p* = 0.37) and negative for StandG (r = − 0.26, *p* = 0.30). A negative correlation was observed between the RPE and HG in SitG (Figure 8E, r = − 0.002, *p* = 0.99), while a positive correlation was found between the RPE and HG in StandG (Figure 8F, r = 0.13, *p* = 0.61).

## 4. Discussion

As was hypothesized, the current study demonstrates the possible benefits of SVT to increase the functionality of COPD individuals.

In the analysis of the acute effects of the SVT on the muscle strength of the COPD individuals, across the HG measures, StandG presented a significant increase (pre session versus post session). However, no significant difference was observed between the two groups.

According to Coelho-Oliveira et al., 2021 [42], SVT probably reduces the muscle activation levels after the SVT session, suggesting that fewer motor units were required to perform the same handgrip activity. However, a study carried out by Strandkvist et al., 2020 [45] evaluated 389 individuals with COPD in a sitting position and concluded that there was no clear association between HG strength and the level of physical activity. Cheung et al., 2013 [46] investigated the relationship of handgrip strength and chronic diseases and multimorbidity in an aged population analyzing data from 748 men and 397 women. The study concluded handgrip strength is associated with multiple chronic diseases in men and women. HG would be a biomarker of multiple physiological systems. Its augmentation may be a feasible strategy to improve general health and decrease likelihood of having multiple chronic diseases and, hence, premature mortality. In addition, handgrip strength appeared to be a more useful marker of multimorbidity than chronological age in men.

Sá-Caputo et al., 2019 [29] concluded an exploratory study with the aim to assess effects of SVT on functional parameters with chronic disease individuals. The hypothesis was that the SVT could improve functionality in these individuals. Corroborating our study, Sá-Caputo et al., 2019 [29] showed a significant difference (*p* = 0.04), with an increase in the HS, found after the SVT protocol.

The relevance of the increase of the HG strength in this current study can be attributed to some physiological results pointed out in some studies involving this same population. Strandkvist et al., 2020 [45] reported that, among men with COPD, HG strength was associated with fatigue independent of physical activity level. Tsuburai et al., 2022 [47] pointed out that HG strength might be more useful for predicting peak inspiratory flow (PIF) than other parameters. Turan et al., 2019 [48] reinforced the idea that HG strength may be used as a measure of muscle performance in COPD exacerbation, especially when the 6-MWD test cannot be performed.

The range of motion of the trunk (trunk flexibility) was also assessed in this current study. Across the ATF, a significant reduction of this distance between the finger to the ground was found in both groups separately, considering the pre session and the post session. Pfeifer et al., 2022 [49] pointed out that flexibility is an important factor for individuals to successfully perform activities of daily living, and decreased flexibility is not only associated with a greater risk of falling, but also difficulty performing body movements.

Cardinale and Bosco, 2003 [50] verified that the muscle activation due to SVT can induce improvements in muscle performance and an increase in flexibility due to exercise that is generated through the vibration produced in VP. In addition, there is strong evidence of the effects of SVT on fitness, including improvements in the flexibility of the lower limbs [38,51,52].

For the population investigated in this current study, an increase in the range of the motion of the trunk (flexibility) assessed by the FTFD [39] can be identified as an acute effect of SVT, helping to improve the functional capacity of COPD individuals, considering the biomechanical limitations in the thoracic region and spine due to pathological pulmonary mechanics of the COPD individuals [53].

The RPE during the ATF presented no significant difference, considering the pre session and the post session. It was not influenced by the session and the SVT can be considered a safe and feasible type of exercise. Furness et al., 2014 [16] also investigated COPD individuals and concluded that SVT was an efficacious mode of exercise for this population. SVT does not negatively affect the exercise tolerance or exacerbate the disease, while concurrently improving the functional performance of the lower limbs. SVT would increase neuromuscular spindle activity, triggering a reflex–stretch response [54] and consequently create a small and rapid change in muscle length [55]. Thus, SVT seems to benefit COPD individuals by improving the functional exercise capacity, without producing adverse effects [56].

Although it is understood that sarcopenia may be present in most of the individuals analyzed, there were no reports of falls or unpleasant symptoms that may have arisen during SVT. The sitting posture proposed in this study and evaluated in other studies involving populations with chronic disorders [42,57] reinforce the relevance of the SVT and it would be recommended that an exercise modality be included in PR.

The reduced functional exercise capacity and musculoskeletal function have been investigated as important extrapulmonary consequences of the COPD, and SVT has been suggested as an intervention with positive impacts in the COPD individuals. Gloeckl et al., 2021 [34] pointed out that the SVT can significantly improve the physical performance in COPD patients. Furness et al., 2014 [16] found positive effects of SVT on muscle strength, physical performance, and quality of life for COPD individuals. It is also reinforced that the SVT did not exacerbate symptoms of COPD that can be associated with physical inactivity. Lage et al., 2018 [58] reported that SVT has gained prominence in the rehabilitation of COPD individuals because it is a safe low-intensity exercise that leads to beneficial effects on physical performance and quality of life.

Exercise training has great potential benefits that can improve functional status [59] of COPD individuals. However, starting a physical program is challenging for patients after an exacerbation, and more time may be needed to find the appropriate exercise protocol for an individual patient [60]. Then, the SVT has been gaining prominence as an exercise modality option in PR [16,17,56].

Considering the correlation analysis between ATF and HG, a positive correlation was found for StandG (both *p* ≤ 0.05), indicating that in this group, the improvement of the range of motion can be associated with better HG parameters; between RPE and ATF (Figure 8C,D) (r = 0.20, *p* = 0.37), a positive correlation was found for SitG, confirming that the perceived effort during the trunk range of motion test did not increase among SitG participants. It is relevant to highlight that all correlations were low or very low.

Still, the performance of the SVT in the sitting posture (SitG) has seemed to be more adequate regarding the RPE, while performing the SVT in the standing posture (StandG) seems to favor the gain of HG strength in the COPD individuals.

### Limitations

The current study has some limitations such as the study design being without a control group. Moreover, the fingertip-to-floor distance was used. However, the sit-and-reach or straight-leg-rise test would be recommended due to their validity and reliability.

## 5. Conclusions

The COPD individuals may benefit from the SVT promoting physiological responses that contribute to improved functional parameters, such as muscle strength and flexibility, in a single session of SVT. It is suggested that WBV exercise would be suitable for COPD individuals and justify the inclusion of this type of exercise in PR protocols.

## Figures and Tables

**Table 1:** Anthropometric characteristics and clinical data of the COPD individuals.

Variables	SitG (*n* = 21)	StandG (*n* = 17)	*p* Value
Age (years)	67.62 ± 6.65	67.82 ± 2.65	*p* ˃ 0.05
Gender (F/M)	9/12	10/7	-----
Height (cm)	160 ± 10.35	150.5 ± 29.56	*p* ˃ 0.05
Body mass (kg)	67.33 ± 17.36	69.38 ± 18.03	*p* ˃ 0.05
BMI (kg/m^2^)	25.81 ± 5.54	26.02 ± 4.93	*p* ˃ 0.05
DMT2	5%	5%	------
SAH	10%	9%	------
Tabagism	60%	70%	------
pSO_2_ (%)	95.95 ± 5.39	98.29 ± 0.58	*p* ˃ 0.05
RR (rpm)	20.11 ± 3.32	19.50 ± 1.77	*p* ˃ 0.05

Sitting group (SitG); Stand group (StandG); Female (F); Male (M); Body mass index (BMI); Diabetes mellitus type 2 (DMT2); Systemic arterial hypertension (SAH); partial oxygen saturation (pSO_2_); RR—Respiratory rate. Data expressed as Mean ± SD.
